# Complete Genome Sequence of Mobuck Virus Isolated from a Florida White-Tailed Deer (Odocoileus virginianus)

**DOI:** 10.1128/MRA.01324-18

**Published:** 2019-01-17

**Authors:** Mohammad Shamim Ahasan, Juan M. Campos Krauer, Kuttichantran Subramaniam, John A. Lednicky, Julia C. Loeb, Katherine A. Sayler, Samantha M. Wisely, Thomas B. Waltzek

**Affiliations:** a Department of Infectious Diseases and Immunology, College of Veterinary Medicine, University of Florida, Gainesville, Florida, USA; b Faculty of Veterinary and Animal Sciences, Hajee Mohammad Danesh Science and Technology University, Basherhat, Bangladesh; c Department of Large Animal Clinical Sciences, College of Veterinary Medicine, University of Florida, Gainesville, Florida, USA; d Department of Wildlife Ecology and Conservation, University of Florida, Gainesville, Florida, USA; e Department of Environmental and Global Health, College of Public Health and Health Professions, University of Florida, Gainesville, Florida, USA; f Emerging Pathogens Institute, University of Florida, Gainesville, Florida, USA; University of Maryland School of Medicine

## Abstract

Here, we report the complete genome sequence of mobuck virus isolated from a Florida white-tailed deer (Odocoileus virginianus) in 2017. This is the second report of mobuck virus in the United States and expands the known geographic range of this novel orbivirus into Florida.

## ANNOUNCEMENT

The family *Reoviridae* is a diverse assemblage of nonenveloped double-stranded RNA viruses, some of which are notable human and veterinary pathogens. The genomes of reoviruses consist of 10 to 12 segments, encoding structural and nonstructural proteins (1, 2). The family consists of 15 genera, including the genus *Orbivirus,* which includes 22 species recognized by the International Committee on Taxonomy of Viruses (3). Orbiviruses are globally distributed, infecting a wide range of vertebrate hosts, including birds, ruminants, horses, marsupials, nonhuman primates, and humans (1). Orbiviruses are transmitted primarily by arthropod vectors such as *Culicoides* midges, sandflies, mosquitos, and ticks (1, 4). Orbiviruses can cause either inapparent infections or high-morbidity/mortality epizootics, depending upon host, viral, and environmental factors (5).

In September 2017, a spleen sample was collected from a dead farmed white-tailed deer (animal no. OV612) that was suspected of having hemorrhagic disease. The presence of epizootic hemorrhagic disease virus (EHDV) genomic RNA in the homogenized spleen specimen was detected by reverse transcription-PCR (RT-PCR) (6) and further suggested by observation of a virus-induced cytopathic effect 9 days postinoculation into Aedes albopictus clone C6/36 (ATCC CRL-1660) cells. The viral RNA was extracted and sequenced on an Illumina MiSeq sequencer as previously described (7). After filtering the low-quality reads and quality trimming in CLC Genomic Workbench v10.1.1 using default parameters, a total of 4,172,407 high-quality reads were obtained with an average read length of 247 bp. Following removal of host (i.e., Aedes albopictus) sequences (GenBank accession no. MNAF00000000) by using Kraken v1.0 (8), *de novo* assembly of 184,810 paired-end reads was performed in SPAdes v3.10.0 with default parameters (9). BLASTX analysis of the 24 resulting contigs (*N*_50_, 405 bp), performed in CLC Genomic Workbench v10.1.1 against a custom virus database created from virus protein sequences retrieved from the UniProt Knowledgebase (https://www.uniprot.org/uniprot/), revealed that EHDV serotype 2 (EHDV-2) and mobuck virus (MBV) were present. The total length of the complete coding sequences of the 10 mobuck virus strain OV612 (MBV-OV612) segments was 18,792 bp (6,264 amino acids [aa]), with a G+C content of 39.50%. After mosquito sequences were removed by Kraken, the quality of the assembly was determined by mapping the reads back to the MBV-OV612 complete coding sequences using Bowtie 2 (10) and visually inspecting the alignments using Tablet (11). The average coverage of the MBV-OV612 genome was 747 reads/nucleotide (nt). MBV-12-2, first isolated from a postmortem spleen sample of a farmed white-tailed deer in Missouri in 2012 (4), displayed a similar total coding sequence length of 18,759 bp and a G+C content of 39.57%. The 5′ and 3′ untranslated regions of the 10 segments of MBV-OV612 were not determined. BLASTN analysis of the 10 MBV-OV612 genome segments against the NCBI database revealed the highest nucleotide identity (78 to 99%) with the prototype MBV-12-2. Maximum likelihood phylogenetic analysis performed in IQ-TREE (12), based on the alignment of the T2 protein amino acid sequences from 29 orbiviruses supported the Florida MBV-OV612 strain being the closest relative to the original Missouri MBV-12-2.

MBV has been proposed as the prototype of a new species in the mosquito-borne clade of orbiviruses (4). Both MBV isolates were recovered from farmed white-tailed deer exhibiting clinical signs typical of epizootic hemorrhagic disease, and both deer were coinfected with EHDV-2, calling into question the role of MBV in the diseased deer. In our study and a previous study (4), MBV was isolated in mosquito cells, which supports its position among the mosquito-borne orbiviruses, a finding that underscores the need to determine the natural mosquito vector(s). Future studies are needed to better define MBV pathogenicity, epidemiology, host range, and potential economic impact on farmed and wild white-tailed deer populations.

### Data availability.

The genome sequences for MBV-OV612 and raw sequence data have been deposited in NCBI GenBank and Sequence Read Archive under accession no. MH801195 to MH801204 and SRP169613, respectively.
